# GABA_A_ receptor modulation by terpenoids from *Sideritis* extracts

**DOI:** 10.1002/mnfr.201300420

**Published:** 2013-11-24

**Authors:** Artur Kessler, Hilal Sahin-Nadeem, Sarah C R Lummis, Ingrid Weigel, Monika Pischetsrieder, Andrea Buettner, Carmen Villmann

**Affiliations:** 1 Department of Chemistry and Pharmacy, Food Chemistry Division, University of Erlangen-NuernbergErlangen, Germany; 2 Department of Food Engineering, Faculty of Engineering, Akdeniz UniversityAntalya, Turkey; 3 Department of Biochemistry, University of CambridgeCambridge, UK; 4 Sensory Analytics, Fraunhofer Institute for Process Engineering and Packaging (IVV)Freising, Germany; 5 Institute for Clinical Neurobiology, Julius-Maximilians-University of WuerzburgWuerzburg, Germany

**Keywords:** GABA_A_ receptor, Patch clamp recordings, Terpene, Two-electrode voltage clamp recordings, Volatile odorants

## Abstract

**Scope:**

GABA_A_ receptors are modulated by *Sideritis* extracts. The aim of this study was to identify single substances from *Sideritis* extracts responsible for GABA_A_ receptor modulation.

**Methods and results:**

Single volatile substances identified by GC have been tested in two expression systems, *Xenopus* oocytes and human embryonic kidney cells. Some of these substances, especially carvacrol, were highly potent on GABA_A_ receptors composed of α1β2 and α1β2γ2 subunits. All effects measured were independent from the presence of the γ2 subunit. As *Sideritis* extracts contain a high amount of terpenes, 13 terpenes with similar structure elements were tested in the same way. Following a prescreening on α1β2 GABA_A_ receptors, a high-throughput method was used for identification of the most effective terpenoid substances on GABA-affinity of α1β2γ2 receptors expressed in transfected cell lines. Isopulegol, pinocarveol, verbenol, and myrtenol were the most potent modifiers of GABA_A_ receptor function.

**Conclusion:**

Comparing the chemical structures, the action of terpenes on GABA_A_ receptors is most probably due to the presence of hydroxyl groups and a bicyclic character of the substances tested. We propose an allosteric modulation independent from the γ2 subunit and similar to the action of alcohols and anesthetics.

## 1 Introduction

Odorants are able to stimulate signaling pathways and some of these compounds are known to potentiate GABA_A_ receptors (GABA_A_Rs) 1–3. *Sideritis* species are the basis for evening tea preparations consumed in Mediterranean countries, with their popularity possibly due to the sedative properties of *Sideritis* extracts 4. The genus *Sideritis*, however comprises more than 150 species. Tea preparations are predominantly used because of their anti-inflammatory, anticonvulsive, and antioxidative properties 4. One of the sedative pathways of *Sideritis* may involve GABA_A_Rs with extracts potentially causing an enhancement of GABA-gated currents similar to the actions of benzodiazepines.

GABA is the major inhibitory neurotransmitter in the central nervous system. GABA-induced activation of ionotropic GABA_A_Rs leads to hyperpolarization of the membrane due to the influx of Cl^−^ ions into the postsynaptic neuron in the adult brain. GABA_A_Rs are not only expressed in a wide variety of neurons in various brain regions, e.g. cortex, hippocampus, cerebellum, and olfactory bulb, but also in non-neuronal cells and peripheral tissues. Due to a large repertoire of identified subunits, there could be a huge variability of subunit combinations forming pentameric GABA_A_R complexes. Most of the heteromeric receptors contain α and β subunits with the α1β2γ2 composition representing the most abundant receptor isoform (around 60% of all GABA_A_Rs) in the brain 5. Distinct subunit combinations have been found to either mediate sedative (e.g. α1β2γ2) or anxiolytic effects (e.g. α2β3γ2) or both 6,7. Furthermore, GABA_A_Rs are important as they bind anticonvulsants and anesthetics. The binding site for diazepam, a highly potent anticonvulsant drug, has been studied intensively and is localized at the interface of the α and the γ2 subunits.

GABA_A_Rs are members of the Cys-loop receptor superfamily, which also includes nicotinic acetylcholine receptors (nAChR), glycine receptors, and the serotonin receptors (5HT_3_R). These all share a similar topological organization with a large extracellular N-terminus harboring a conserved disulfide bridge forming the eponymous Cys-loop. The N-terminus carries 10 β-sheets in an Ig-like structural organization and forms the orthosteric binding site, which is located between adjacent subunits of the pentameric receptor complexes 8. Site-directed mutagenesis studies have provided much information about this binding site, as well as binding sites for the many agents that can modulate the function of these receptors 9,10.

Recently, it was shown that these receptors are also a target of volatile odorant extracts 3, and actions on GABA_A_Rs were described for substances from the essential oils such as geraniol or linalool 11. A subunit-specific action of fragrant dioxane derivatives was demonstrated on β1-containing GABA_A_Rs, providing a novel tool to detect β1-containing neurons e.g. in neurons of the hypothalamus 12.

Odorants bind to odorant receptors at the olfactory epithelium 13, but it is not yet clear how they reach the brain. The axons of the olfactory sensory neurons could send information to second-order neurons in the olfactory bulb or these compounds could simply diffuse through lipid membranes due to their small size and high hydrophobicity 14,15. Diazepam, for example, is able to pass the blood–brain barrier due to its hydrophobic nature 16. An in vivo study has demonstrated that inhalation of essential oil extracted from *Abies sachalinensis* resulted in much higher concentrations of odorant compounds in the brain when compared to injection into the peritoneum 17. Nevertheless, more detailed studies are required to solve this issue. Another behavioral study with the species *Sideritis clandestina* demonstrated the anxiolytic and antioxidant potential of plant extracts. Mice that had ad libitum access to *S. clandestina* tea over a period of 6 weeks showed decreased thigmotaxis time, an enhanced number of entries into a central area, and enhanced levels of reduced glutathione, a marker protein for antioxidant capacity 18.

Here, we have analyzed several odorant compounds originally identified in *Sideritis* species. Using GC analysis, we were able to show single components of these extracts and tested them separately on α1β2γ2 or α1β2 receptors expressed either in HEK293 cells or in *Xenopus* oocytes. Some terpenes, and also various terpene-derived compounds potentiated the GABAergic responses. A search for other terpenes with structural similarities revealed 13 additional candidates. A detailed study of the functional effects of these compounds has allowed us to identify, which structural features are important for their modulatory properties.

## 2 Materials and methods

### 2.1 Chemicals

GABA ≥ 99%, DMSO ≥ 99.9%, 1-Octen-3-ol ≥ 98%, Carvacrol ≥ 98%, l-Carveol (mixture of cis and trans) ≥ 95%, Isopulegol ≥ 99%, Linalool ≥ 97%, Myrtenol ≥ 95%, trans-Pinocarveol ≥ 96%, (S)-cis-Verbenol ≥ 95%, α-Pinene ≥ 98%, β-Pinene ≥ 99%, β-Caryophyllene ≥ 80%, β-Myrcene ≥ 95%, Caryophyllene oxide ≥ 95%, α-Terpineol ≥ 96%, and all other chemicals were purchased from Sigma-Aldrich (Taufkirchen, Germany). Dichloromethane (DCM) was obtained from Acros (Geel, Belgium) and freshly distilled before use. Reference compounds for GC-analyses were diluted with DCM to an appropriate concentration. Stock solutions of compounds at a concentration of 1 M were prepared using DMSO and dissolved in the appropriate buffer, prior to use. All reservoirs for control and compound application in electrophysiological recordings contained equivalent amounts of DMSO. The solvent showed no effect on GABA-mediated responses up to 0.1% concentration (data not shown).

### 2.2 GC-MS

#### 2.2.1 Preparation of Sideritis extracts

Extracts containing the volatile fraction of an infusion of *Sideritis* were prepared as follows: 5 g of crushed plant material was added to 150 mL of boiled water and stirred at 70°C for 1 h. After filtration and cooling to room temperature, the infusion was mixed with 50 mL DCM and the volatile fraction was separated via solvent assisted flavor evaporation 19. After separation, the organic phase was dried over anhydrous Na_2_SO_4_ and concentrated using a Vigreux column and microdistillation 20. The extract was reduced to a final volume of 200 μL. Samples of *Sideritis arguta*, *Sideritis condensata*, *Sideritis stricta*, and *Sideritis sipylea* were extracted and diluted to an appropriate content with DCM, before analyses.

#### 2.2.2 GC-MS

Analyses were performed on a Thermo Trace GC Ultra (Thermo Scientific, S.p.A., Rodano, Italy) coupled with an ITQ 900 IT mass spectrometer. Analytical capillaries used were DB5 and DB-FFAP (J&W Scientific, Fisons Instruments, Mainz, Germany), in the dimension of 30 m × 0.25 mm × 0.25 μm. The GC temperature program started from initial 40°C, held for 7 min and increasing up to 250°C at a rate of 8°C/min, held for 15 min. Helium was used as carrier gas at constant flow of 0.8 mL/min and the injected sample volume was 2 μL. The temperature was set to 250°C for the transfer line and to 200°C for the ion source. MS detection mode was EI with a current of 70 eV (Full scan, *m/z* 30–300). Identification of substances was achieved comparing the obtained mass spectra and retention indices in the samples to the spectra of reference substances injected in parallel 21. Separation was performed on two different analytical gas chromatographic phases.

### 2.3 Electrophysiology

#### 2.3.1 HEK293 cell preparation

HEK293 cells (Clontech, Saint-Germain-en-Laye, France) were grown in Modified Eagle Medium with Earle's Salts (MEM; PAA, Pasching, Austria), supplemented with 10% fetal calf serum, l-glutamine (200 mM), and 50 U/mL penicillin and streptomycin at 37°C and 5% CO_2_. HEK293 cells were transiently transfected using the Superfect transfection reagent (Qiagen, Hilden, Germany). Rat receptor cDNA (kindly provided by Proffesor W. Sieghart, Vienna) was used to express a receptor composition of α1β2γ2L subunits. The cDNA stoichiometry was 1:1:2 α1β2γ2L to ensure the incorporation of gamma subunits into the receptor complex. To visualize transfected cells, cDNA encoding for green fluorescent protein was cotransfected.

#### 2.3.2 Whole-cell patch clamp technique

Current amplitudes were measured by the patch clamp technique in a whole-cell configuration. Current signals were amplified with an EPC-9 amplifier (HEKA, Goettingen, Germany). After transfection (24–48 h), whole-cell recordings from HEK293 cells were performed by application of ligand using a U-tube. The external buffer consisted of 137 mM NaCl, 5.4 mM KCl, 1.8 mM CaCl_2_, 1 mM MgCl_2_, 5 mM HEPES, pH adjusted to 7.2 with NaOH; the internal buffer was 120 mM CsCl, 20 mM N(Et)_4_Cl, 1 mM CaCl_2_, 2 mM MgCl_2_, 11 mM EGTA, 10 mM HEPES, pH adjusted to 7.2 with CsOH. Recording pipettes were fabricated from borosilicate capillaries with an open resistance of 3–6 MΩ. Current responses were measured at a holding potential of −60 mV. The ligand GABA was dissolved in extracellular buffer. Substances alone solved in DMSO and diluted in extracellular solution did not elicit a current response (data not shown). Similarly, untransfected cells did neither respond to GABA or to GABA plus modulator (data not shown).

#### 2.3.3 Oocyte preparation

Following surgical preparation of the oocytes from the ovaries of *Xenopus laevis*, oocytes were isolated by enzymatic digestion at 19°C for 3–4 h with 600–700 U/mL type 2 collagenase from *Clostridium histolyticum* (CLS 2, Worthington, Lakewood, NJ, USA) dissolved in OR2 solution containing 82.5 mM NaCl, 2 mM KCl, 1 mM MgCl_2_, and 1 mM HEPES (pH 7.4 with Tris). Defollicularized oocytes were obtained from the Institute of Cellular and Molecular Physiology (University of Erlangen-Nuernberg, Germany). The cDNAs of rat α1 and β2 GABA_A_ subunits were subcloned into the pSGEM vector. Plasmids encoding for the human α1, β2, and γ2 subunits were provided by Bjarke Ebert, Lundbeck (Valby, Denmark) and directly used for in vitro transcription. The corresponding cRNA was synthesized with the help of a mMessage mMachine T7 RNA polymerase kit (Ambion, Austin, TX, USA), following the manufacturer's protocol. To express a functional GABA_A_R, 1.2–2.4 ng of a 1:1 mixture of rat α1 and β2 receptor subunit cRNA was injected using a Nanoject II injector (Drummond Scientific, USA). The human α1β2γ2 receptor configuration was expressed by injecting 1.56–3.12 ng of a 1:1:3 mixture of the corresponding cRNA. The oocytes were incubated in ND96 buffer (96 mM NaCl, 2 mM KCl, 1 mM MgCl_2_, 1 mM CaCl_2_, 5 mM HEPES, pH adjusted to 7.4 with NaOH) at 16–18°C for 24 h, before use.

#### 2.3.4 Electrophysiological recordings

The oocytes were used after a 24 h incubation period. Activity of GABA_A_Rs was measured with the two-electrode voltage clamp technique. Recording pipettes (GB 150F-10, Science Products, Hofheim, Germany) were manufactured with a P97 micropipette puller (Sutter Instruments, USA) and filled with 3 M KCl to give a resistance from 0.2–2 MΩ. Oocytes were clamped at −50 mV using a Turbo Tec-03x npi amplifier (npi electronic GmbH, Tamm, Germany) and constantly superfused with ND96 during recording. Current traces were recorded at 300 Hz and filtered at 200 Hz using cell works software.

The concentration for GABA used in these experiments was 1 μM corresponding to an EC_5–10_, as it has been previously shown that the use of a low GABA concentration is required to observe small effects by the modulators 2,11. Modulators were tested using GABA plus increasing concentrations of compound and with a control (GABA only) response after every compound application. Washout time between measurements was at least 3 min and was prolonged to 6 min for the highest concentrations. To exclude nonspecific measurements, only reversible effects were taken into account. Recordings were repeated with at least three oocytes from two independent batches. All experiments were carried out at room temperature (∼22°C).

### 2.4 Flexstation experiments

#### 2.4.1 Cell culture and preparation

HEK293 cells were grown on 90 mm tissue culture plates containing DMEM/Glutamax medium (Invitrogen, Paisley, UK), supplemented with 10% fetal calf serum, at 37°C and 5% CO_2_ in a humidified atmosphere. Cells were transiently transfected using polyethyleneimine (PEI, 25 kDa, linear, Polysciences, Eppelheim, Germany). For this purpose, 5 μg of human α1 and β2 were diluted together with 10 μg of γ2 subunit DNA in 1 mL of serum-free medium followed by the addition of 90 μL PEI (1 mg/mL). After 10 min incubation at room temperature, the mixture was added dropwise to a 80–90% confluent plate, and incubated for 24 h. Cells were transferred to black 96-well plates (Greiner Bio-One, Stonehouse, UK), which were pretreated with 0.01% Cultrex poly-l-Lysine (Trevigen, Gaithersburg, MD, USA). After an additional 24 h, incubation cells were used for experiments.

#### 2.4.2 Dye loading and compound preparation

Cells were washed twice with FLEX buffer (115 mM NaCl, 1 mM KCl, 1 mM MgCl_2_, 1 mM CaCl_2_, 10 mM HEPES, 10 mM D-glucose, pH adjusted to 7.2 with NaOH). A total of 100 μL fluorescent membrane potential (FMP) blue dye (FLIPR Membrane Potential Blue Kit, Molecular Devices, Wokingham, UK) in Flex buffer (dilution 1:1000) was then added to each well. In case of modulation experiments, the appropriate concentrations of the substances were directly added with dye to the plates. The plates containing the transfected cells and the fluorescent dye (± a distinct concentration of modulatory substance) were incubated for 30 min at 37°C before starting the measurement. Then, another plate containing the appropriate GABA concentrations diluted in FLEX buffer was prepared.

#### 2.4.3 Fluorometric assay

Dye-loaded cells (± a distinct concentration of modulatory substance) and the plate containing various GABA concentrations were transferred to the FlexStation II Device (Molecular Devices). Fluorescence measurements were performed using Softmax Pro software. Before GABA was applied, a baseline was recorded for 20 s. Each well was read five times with high PMT adjustment. Fluorescence values were processed using a peak kinetic reduction algorithm.

### 2.5 Analysis of data

Fluorescence data were normalized and the dose-response curves were generated using Prism 4 (GraphPad Software) with a sigmoidal dose response (variable slope) equation. Statistical analysis was performed using GraphPad InStat 3.0 (Graphpad Software). Comparisons with control application were made with one-way ANOVA method and Dunnett post hoc test. Within a particular concentration, the Tukey post hoc test was used to determine substance specific effects. *p*-values of significance were indicated in the appropriate figures and within the text.

## 3 Results

**Figure 1:**
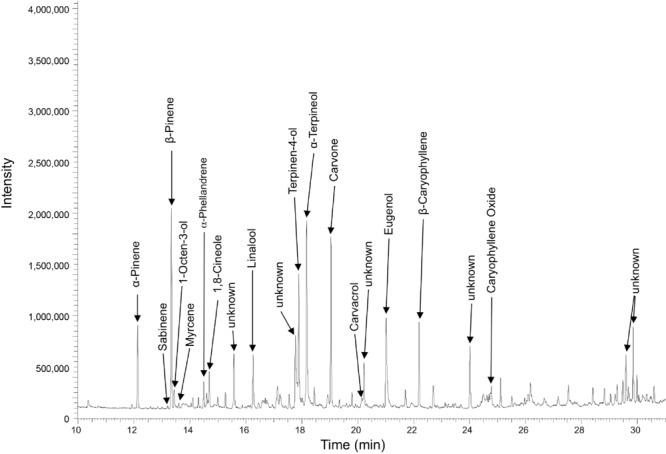
Representative gas chromatographic pattern (GC-flame ionization detector, analytical column DB5) of a *Sideritis stricta* extract for identification of single substances as candidates for GABA_A_R modulation.

Single components of various *Sideritis* extracts were analyzed using GC to identify possible candidates for GABA_A_Rs modulation (Fig.1). These extracts are rich in terpenes. Specifically, the compounds α- and β-pinene, terpinen-4-ol, α-terpineol, β-caryophyllene and its oxide, sabinene, α-phellandrene, linalool, eugenol, carvone, 1,8-cineole, 1-octen-3-ol, and carvacrol were, among others, found as quantitatively dominant or clearly detectable compounds characteristic for the investigated *Sideritis* varieties (Fig.1). The following substances were selected for further investigation in physiological studies: α-pinene and β-pinene, sabinene, α-phellandrene, and β-caryophyllene, linalool, 1-octen-3-ol, and carvacrol. Among these candidates are not only pure terpenes and terpene hydroxyl derivatives, but also substances that represent different chemical groups, e.g. 1-octen-3-ol as aliphatic alcohol, β-caryophyllene as sesquiterpene, and carvacrol as monoterpenoid phenol.

**Figure 2:**
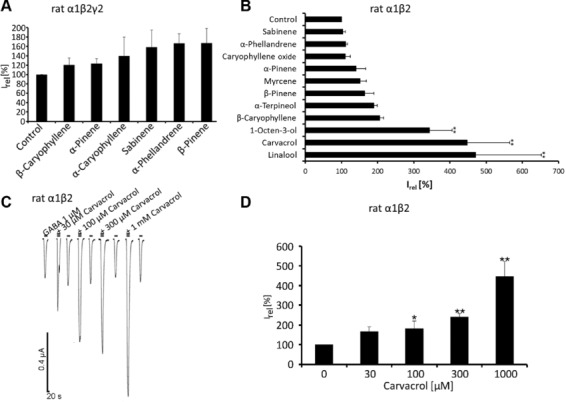
GABA_A_R modulation by *Sideritis* components. (A) Comparison of whole-cell currents (I_abs_) following GABA (1 μM) application ± *Sideritis* components especially various pinenes at 1 mM from HEK293 cells expressing rat α1β2γ2 GABA_A_Rs. Data are mean current values ± SD, mean *I*_abs_ of GABA application alone = 100%, *n* = number of measured cells; *n* = 4–8. (B) Similar experiment to (A) but recordings were performed using *Xenopus* oocytes expressing rat α1β2 GABA_A_Rs. GABAergic currents ± single components from *Sideritis* extract at 1 mM, GABA = 1 μM concentration, *n* = 4–8 (C) Representative traces following GABA coapplication with the most potent substance carvacrol (range from 30 μM–1 mM). (D) Bar diagram demonstrating the mean current amplitudes for the potentiation of GABAergic currents by different carvacrol concentrations. Mean absolute current values are shown ± SD, *n* = 3–6. The significance of the depicted currents were compared to control (GABA application alone), **p < 0.01 with Dunnett post hoc test.

These substances were tested for their potentiating effect on GABA-evoked currents in injected *Xenopus* oocytes and transfected HEK293 cells expressing α1β2 or α1β2γ2 GABA_A_Rs (Fig.2). The presence of the γ2 subunit was tested using a coapplication of Zn^2+^, which only inhibits α1β2 receptors (data not shown) 22, although recordings from both cell types yielded similar results arguing for an effect independent from the γ2 subunit. Application of compounds in the absence of GABA did not elicit any currents (data not shown). Pinenes had little or no modulatory effects; the current values using high concentrations (1mM) of either α-pinene or β-pinene showed only a small increase in the GABA-evoked responses of 0.35 ± 0.08 μA (compared to 0.25 ± 0.08 μA without α-pinene) and 0.35 ± 0.08 μA (compared to 0.24 ± 0.06 μA without β-pinene), in *Xenopus* oocytes and 1.37 ± 0.66 nA for α-pinene compared to 1.18 ±. 0.58 nA without modulator, and 0.52 ± 0.16 nA for β-pinene compared to 0.29 ± 0.07 nA for GABA alone in transfected HEK293 cells (Table1; see relative I-values of 1 μM GABA). Similarly, substances such as sabinene, α-phellandrene, and β-caryophyllene did not enhance responses (Fig.2B). A significant potentiation, however, was observed for 1-octen-3-ol, linalool, and carvacrol (Fig.2B). Therefore, carvacrol was tested in a dose-dependent manner using concentrations from 30 μM to 1 mM (Fig.2C). The relative current amplitudes compared to GABA alone showed that carvacrol at concentrations of 100 μM (1.8 fold potentiation) or greater significantly enhanced GABAergic currents (up to 4.5 fold, Fig.2D). Thus, carvacrol was among the substances identified from *Sideritis*, the most potent modulator on GABA_A_Rs comparable to linalool and 1-octen-3-ol (Fig.2B) 11.

**Table 1:** Current amplitudes of ionotropic GABA_A_Rs modulated by odor substances

Expressed subunits	Expression system	GABA [μM]	*I*_abs_ [μA]	Modulator	GABA + modulator [μM]	*I*_abs_ [μA]	I_rel_ [% of 1 μM GABA]	*n*	Originally identified in *Sideritis* extract
Rat α1β2	*Xenopus* oocytes								
		1	0.41 ± 0.07	α-Phellandrene	1000	0.46 ± 0.08	112 ± 19	3	^*^
		1	0.24 ± 0.06	β-Pinene	1000	0.35 ± 0.08	134 ± 31	7	^*^
		1	0.26 ± 0.05	Linalool	1000	1.23 ± 0.25	473 ± 96	4	^*^
		1	1.99 ± 0.64	1-Octen-3-ol	1000	6.40 ± 1.74	321 ± 87	4	^*^
		1	0.58 ± 0.13	Carveol	600	1.71 ± 0.26	294 ± 45	10	
		1	0.31 ± 0.10	Pinocarveol	600	1.11 ± 0.32	358 ± 103	7	
		1	0.30 ± 0.04	Isopulegol	600	1.25 ± 0.43	416 ± 143	8	
		1	0.18 ± 0.09	Carvacrol	300	0.44 ± 0.22	414 ± 142	5	^*^
		1	0.55 ± 0.25	Myrtenol	300	2.55 ± 1.00	463 ± 181	7	
		1	0.38 ± 0.16	Verbenol	300	1.00 ± 0.38	263 ± 99	7	
		1	0.25 ± 0.08	α-Pinene	1000	0.35 ± 0.08	140 ± 32	7	^*^
		1	0.23 ± 0.05	Myrcene	1000	0.34 ± 0.06	147 ± 26	7	^*^
		1	0.22 ± 0.04	Caryophyllene oxide	1000	0.24 ± 0.04	109 ± 18	7	^*^
		1	0.052 ± 0.029	Sabinene	1000	0.057 ± 0.034	109 ± 65	4	^*^
		1	0.04 ± 0.02	β-Caryophyllene	1000	0.085 ± 0.04	212 ± 99	4	^*^
		1	0.09 ± 0.04	α-Terpeniol	1000	0.16 ± 0.06	177 ± 66	3	^*^
		1	0.51 ± 0.09	Cedrol	600	1.09 ± 0.18	213 ± 35	11	
		1	0.53 ± 0.09	Nerolidol	600	0.73 ± 0.11	137 ± 21	11	
		1	0.46 ± 0.28	2-Methyl-3-buten-2-ol	600	1.19 ± 0.28	258 ± 61	8	
		1	0.59 ± 0.13	β-Citronellol	600	0.99 ± 0.18	167 ± 30	10	
		1	0.26 ± 0.05	α-Santonin	600	0.57 ± 0.12	219 ± 46	3	
		1	0.31 ± 0.09	Theaspiran	600	0.68 ± 0.15	219 ± 48	7	
		1	0.84 ± 0.27	α-Thujone	600	0.52 ± 0.20	62 ± 24	3	
		1	0.33 ± 0.06	Terpinolene	600	0.69 ± 0.16	209 ± 48	6	
		1	0.37 ± 0.07	Myrtenal	600	0.89 ± 0.18	240 ± 48.5	8	
		1	0.31 ± 0.02	Nootkatone	600	0.46 ± 0.06	148 ± 19	7	
Human α1β2γ2	*Xenopus* oocytes								
		1	0.79 ± 0.25	Linalool	300	1.69 ± 0.84	213 ± 105	10	
		1	0.22 ± 0.05	1-Octen-3-ol	300	0.65 ± 0.11	295 ± 50	6	
		1	0.13 ± 0.08	Carveol	300	0.59 ± 0.23	453 ± 176	8	
		1	0.35 ± 0.08	Pinocarveol	300	1.67 ± 0.24	477 ± 68	8	
		1	0.49 ± 0.13	Isopulegol	300	1.67 ± 0.035	340 ± 70	7	
		1	1.72 ± 0.64	Carvacrol	300	3.86 ± 1.46	224 ± 85	6	
		1	0.35 ± 0.12	Myrtenol	300	2.58 ± 0.82	737 ± 234	7	
		1	0.11 ± 0.01	Verbenol	300	0.89 ± 0.13	809 ± 118	6	
Human α1β2γ2	*HEK293*		I_abs_ [nA]			I_abs_ [nA]			
		1	0.51 ± 0.15	α-Phellandrene	1000	0.83 ± 0.21	168 ± 42	3	^*^
		1	0.29 ± 0.07	β-Pinene	1000	0.52 ± 0.16	179 ± 55	4	^*^
		1	1.18 ± 0.58	α-Pinene	1000	1.37 ± 0.66	116 ± 56	3	^*^
		1	0.23 ± 0.04	Sabinene	1000	0.36 ± 0.06	156 ± 26	6	^*^
		1	1.14 ± 0.62	α-Caryophyllene	1000	1.34 ±0.66	117 ± 57	7	^*^
		1	1.32 ± 0.61	β-Caryophyllene	1000	1.53 ± 0.69	115 ± 52	3	^*^

**Figure 3:**
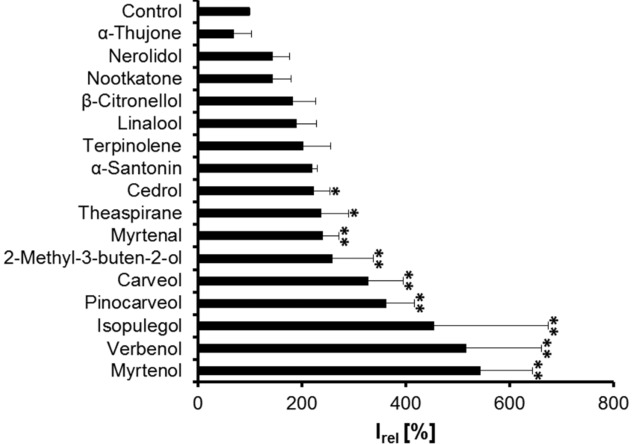
Modulatory effects of terpenoid structures. Currents elicited using two-electrode voltage clamp technique recordings on rat α1β2 expressing oocytes by coapplication of GABA (1 μM) and various terpenoid substances with structural similarities to single components of *Sideritis* extracts. Substance concentration was 600 μM. Mean current amplitudes compared to GABA application alone (100%) ± SD values, *n* = 3–8, significance value **p < 0.01.

To determine, if there are structural motives within the terpene family that may account for the observed modulatory effects on GABA_A_Rs, we probed the effects of 13 structurally related terpenes (Table2). Positive modulation was observed in almost all cases when the substances contained hydroxyl groups, and was enhanced when these substances showed a mono- or bicyclic character (Table1). GABA-evoked responses were significantly potentiated up to 417% by 600 μM isopulegol with I_abs_ values of 1.25 ± 0.43 μA compared to 0.3 ± 0.04 μA in the absence of the volatile compound (Fig.3, Table1). Less effective were cedrol, theaspirane, myrtenal, 2-methyl-3-buten-2-ol, carveol, and pinocarveol, which resulted in 2- to 3.8-fold potentiation (Fig.3, see Table1 for absolute current values). Substances that did not potentiate responses included α-thujone, nerolidol, nootkatone, β-citronellol, terpinolene, and α-santonin, most of which lack a hydroxyl group (Fig.3, Tables1 and 2).

**Table 2:** Terpenoid substances and their structural features used to study modulatory effects on GABA_A_Rs

Compound	Occurence	Class/functional group	Structure
2-Methyl-3-buten-2-ol	Hop	Hemi terpene Hydroxy	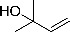
β-Citronellol	Geranium	Acyclic monoterpene Hydroxy	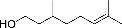
Terpinolene	Scots pine	Monocyclic monoterpene	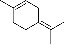
Carveol	Spearmint	Monocyclic monoterpene Hydroxy	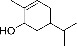
Isopulegol	Lemon eucalyptus	Monocyclic monoterpene Hydroxy	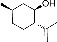
α-Thujone	Artemisia	Bicyclic monoterpene Ketone	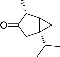
Myrtenal	Cumin seed	Bicyclic monoterpene Aldehyde	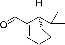
Pinocarveol	Common gum sed	Bicyclic monoterpene Hydroxy	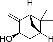
Nerolidol	Neroli	Sesquiterpene Hydroxy	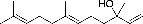
Cedrol	Cedar wood	Sesquiterpene Hydroxy	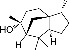
α-Santonin	Artemisia	Sesquiterpene Ketone	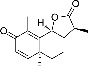
Nootkatone	Grapefruit	Sesquiterpene Ketone	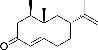
Theaspirane	Wine	Sesquiterpene	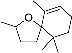

**Figure 4:**
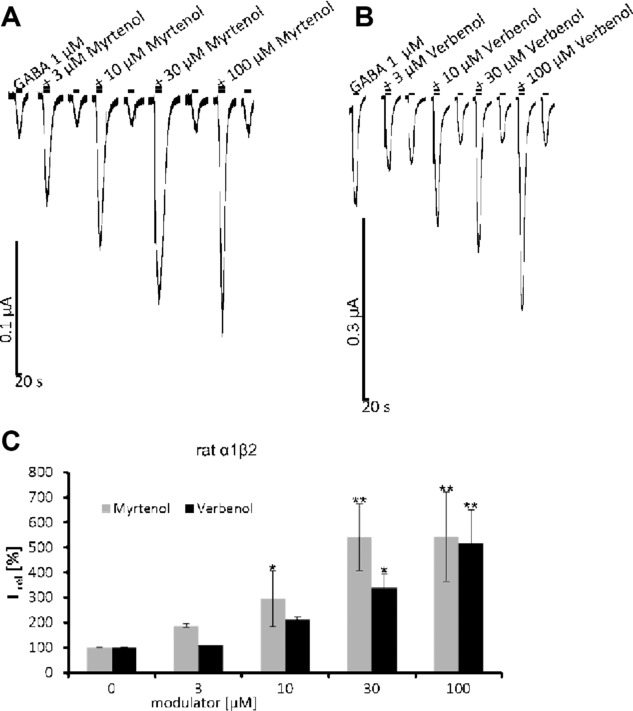
Potentiating effect on GABAergic responses by metabolites of pinenes. Typical traces recorded from oocytes expressing rat α1β2 GABA_A_Rs and activated by GABA (1 μM) plus (A) myrtenol, and (B) verbenol at various concentrations (3 μM–100 μM). (C) Bar diagram representing the dose-dependent potentiation of GABA-mediated currents by verbenol (black bars) and myrtenol (gray bars). Data = mean ± SD, *n* = 3–9. The significance was tested compared to control (GABA alone), *p< 0.05; **p < 0.01.

In addition to the 13 candidates resulting from a comparison of terpene structures, pinene metabolites were also investigated as pinenes were identified in all *Sideritis* extracts analyzed. Previous studies have shown that terpene substances may undergo major biotransformatory processes in vivo so that the active compounds might be represented by the formed derivatives 23. Interestingly, pinene metabolites verbenol and myrtenol at 100 μM were able to enhance GABA-evoked current responses up to 493 ± 82% (verbenol) and 541 ± 94% (myrtenol) of the current magnitudes of the pinene metabolite used compared to GABA application alone (Fig.3). Significant increases of the GABA-mediated currents were also observed at lower concentrations, e.g. 10 μM for myrtenol and 30 μM for verbenol (Fig.4A–C).

**Figure 5:**
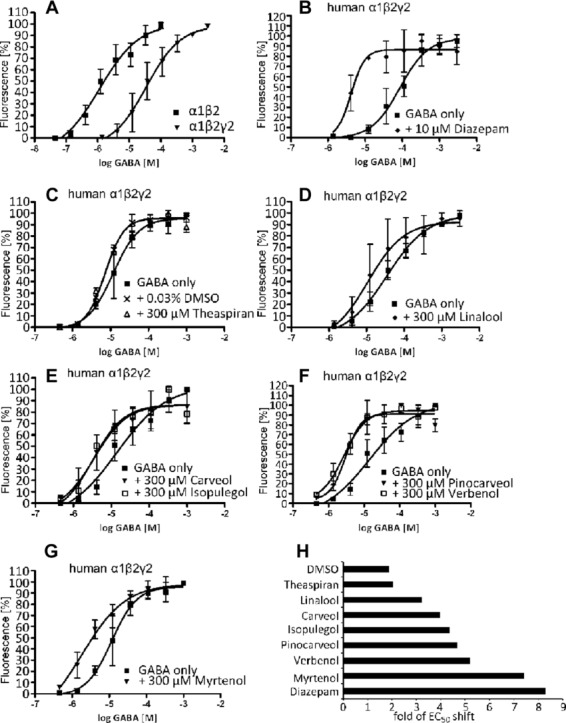
Flexstation data for the most potent modulators. (A) Concentration response curves for human α1β2 and human α1β2γ2 GABA_A_Rs results in a decrease in GABA EC_50_. (B–G) Dose–response curves with either GABA alone (used at concentrations 0.46 μM–1000 μM) or in the presence of different modulators (used at a fixed concentration of 300 μM). Data = mean ± SD, *n* = 3. (H) Bar diagram representing the shift in GABA-affinity (fold in EC_50_ shift).

Potent substances were tested in a high-throughput assay using a Flexstation device. Here, transfected HEK293 cells expressing the α1β2γ2 configuration of the human GABA_A_Rs were used. GABA EC_50_s were 1.4 μM for α1β2 and 20.8 ± 7.0 μM for α1β2γ2 GABA_A_Rs, comparable to previously published data (e.g. Boileau et al.) (Fig.5A) 24. A coapplication of diazepam (10 μM) led to a 8.3-fold decrease in GABA EC_50_ (Fig.5B, Table3). Coapplication of terpenes (300 μM) myrtenol, verbenol, pinocarveol, and isopulegol together with increasing GABA concentrations caused a decrease in GABA EC_50_ (Fig.5). Myrtenol was identified as the volatile compound with the most pronounced effect on the GABA-dose–response curve (7.4 fold decrease in GABA EC_50_)_,_ not dissimilar to the effect of diazepam (Table3), while the decrease was ∼ fourfold for carveol and linalool (Fig.5D, E, and H).

**Table 3:** Affinity changes of GABA EC_50_ values obtained from FLIPR experiments

GABA_A_R subunits transfected	Agonist / modulator	pEC_50_ [M]	EC_50_ [μM]	Ratio [EC_50_ / EC_50_mod]
Human α1β2γ2	GABA	4.75 ± 0.21	20.80 ± 7.00	
	Diazepam	5.37 ± 0.06	4.19 ± 0.86	8.29
	Linalool	4.96 ± 0.38	10.80 ± 0.41	3.22
	Carveol	5.45 ± 0.22	3.59 ± 0.60	3.96
	Isopulegol	5.49 ± 0.16	3.26 ± 0.69	4.36
	Pinocarveol	5.52 ± 0.06	3.03 ± 0.87	4.68
	Verbenol	5.56 ± 0.12	2.73 ± 0.76	5.21
	Theaspirane	5.18 ± 0.08	6.56 ± 0.83	2.06
	Myrtenol	5.73 ± 0.33	1.83 ± 0.46	7.40

Mean values derived from Flexstation measurements (±SD). EC50 values from GABA_A_Rs expressed in HEK293 cells from three independent experiments.

**Figure 6:**
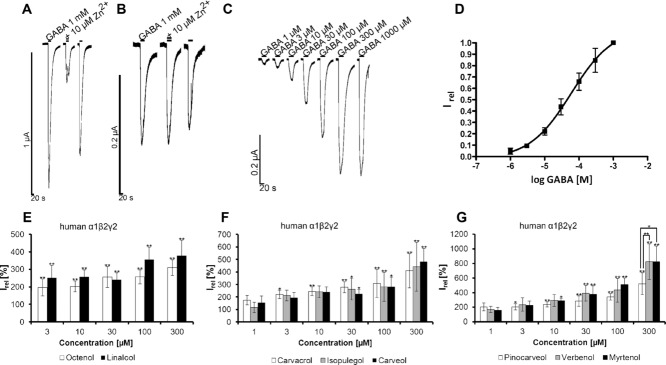
Modulatory effects of volatile terpenoid structures are independent of the origin of GABA_A_Rs subunits. (A) Typical traces of Zn^2+^-mediated inhibition of GABAergic currents on human α1β2 GABA_A_Rs expressed in *Xenopus* oocytes. (B) No Zn^2+^ inhibition was observed when human α1β2γ2 GABA_A_ subunits are expressed. (C) Typical traces to application of various GABA concentrations measured from human α1β2γ2 GABA_A_R expressing oocytes (D) GABA dose–response curve of the human α1β2γ2 GABA_A_R expressed in *Xenopus* oocytes. Data = mean ± SD, *n* = 5; EC_50_ for GABA 59 ± 1.2 μM, the Hill coefficient *n*_H_ was determined to 0.7. (E–G) Dose-dependent modulation of GABAergic currents by various terpenoids carvacrol, carveol, pinocarveol, isopulegol, myrtenol, and verbenol as well as linalool and 1-octen-3-ol as controls. *p < 0.5; **p < 0.01.

We also demonstrated that the effects of the terpenes and terpene-derived substances are similar in rat and human GABA_A_R subunits. Human α1β2γ2 GABA_A_Rs were expressed in oocytes and successful incorporation of the γ2 subunit demonstrated using Zn^2+^: no block was obtained, in contrast to Zn^2+^ inhibition for the α1β2 configuration (Fig.6A–D) 22. Linalool and 1-octen-3-ol (1000 μM) enhanced GABA-evoked currents of α1β2 GABA_A_Rs (Fig.2B). The coexpression of the γ2 subunit did not lead to significant changes in the effectiveness of GABA-current modulation by either linalool or 1-octen-3-ol (300 μM) (Fig.6E). These data indicate that the action of the volatile substances was independent of the γ2 subunit. The effectiveness of other substances and pinene metabolites on human GABA_A_Rs in an α1β2γ2 configuration was similar to the effects observed on rat α1β2 GABA_A_Rs in oocytes and Flexstation data using human α1β2γ2 in HEK293 cells. Modulation by pinocarveol, myrtenol, and verbenol achieved significance when used at concentrations >10 μM whereas for carvacrol, isopulegol, and carveol concentrations of at least 30 μM were required (Fig.6F and G). To determine the modulatory potency of these substances, however, concentration–response curves are required. The potentiating effect of structurally similar volatile substances is, therefore, independent from the origin of the GABA_A_R subunits. Thus, these substances most probably interact with conserved sequence motifs of the α1 and/or β2 GABA_A_R subunits.

## 4 Discussion

Tea preparations from various *Sideritis* species are used as evening tea in Mediterranean countries. Previously, it was shown that mice with ad libitum access to *Sideritis* tea, improved in anxiolytic tests as well as showed enhanced antioxidative capacity in various brain structures. Various nonvolatile compounds were identified that may account for the observed effects 18. Moreover, it was shown that the volatile fraction of *Sideritis* extracts modulates α1β2 and α1β2γ2 GABA_A_R subtypes 3,18. Therefore, the action of the VOCs present in *Sideritis* extracts on GABA_A_R may be a distinct part of the underlying mechanism for a sedative action of such tea preparations in addition to effects of nonvolatile compounds. The volatile extracts potentiated GABAergic responses similar to *Lavender* extracts, where the main component linalool modulates GABA_A_R as well as other receptors such as voltage-dependent calcium channels 11,25–27. In the present study, single components identified within various *Sideritis* extracts have been analyzed for their ability to modulate different GABA_A_R subtypes. The extracts are rich in various terpenoids or terpene-derived structures including carvacrol and pinenes. Carvacrol was identified to significantly enhance α1β2 GABA_A_Rs with an effectiveness comparable to 1-octen-3-ol and linalool, which have been previously shown to modulate GABA_A_Rs 28. Earlier studies on α-pinene and its modulatory effect on GABA_A_Rs expressed in *Xenopus* oocytes have demonstrated an increase in the GABA-evoked currents at millimolar concentrations 29. Our results show that the single substances α-pinene, β-pinene, and β-caryophyllene led to a moderate enhancement of GABA-gated responses on GABA_A_ α1β2 receptors similar to potentiation of the whole *Sideritis* extracts 3. Other substances such as sabinene, α-phellandrene, and caryophellene oxide had no influence on GABAergic currents. However, a combinatorial effect of substances in the extract was not investigated and can, therefore, not be excluded.

Pinenes and other monoterpenes are easily metabolized, e.g. by allylic oxidation in drosophila, rodents, and in humans. The effects of such secondary metabolites on the human central nervous system might be due to molecular and biochemical similarities 30,31. We observed that the substances, which harbored modulatory potential at GABA_A_Rs shared structural similarities such as their cyclic character (mono- or bicyclic), and the presence of a hydroxyl group. Accordingly, 13 similar terpenoid structures have been analyzed as individual candidates. Seven of these showed a strong modulatory effect on GABA_A_Rs of the α1β2 subtype. The same substances acted similarly on GABA-induced currents on α1β2γ2 GABA_A_Rs. A structural comparison revealed that almost all substances that lack modulatory potential did not contain a cyclic structure or they lacked a hydroxyl group. Recently, it was hypothesized that the adjacency of an isopropyl group to the hydroxyl group on a ring structure as present in propofol and menthol might share similar modulatory sites at GABA_A_Rs 32. Similar to the data obtained from *Sideritis* extracts, data on single substances indicate that the modulation is independent from the presence of the γ2 subunit 3. The γ2 subunit is important for the formation of the benzodiazepine-binding site between the α1 and the γ2 subunit. Benzodiazepines such as diazepam are anticonvulsant drugs used to treat patients suffering from various forms of epilepsy 33. Diazepam is, however, structurally dissimilar from terpene structures. Therefore, a modulatory action independent of the γ2 subunit, i.e. via a different binding interface than diazepam, is not surprising 34,35. The X-ray structure of a GABA_A_R homolog from prokaryotes ELIC provided evidence for two distinct binding sites for benzodiazepines an intrasubunit and an intersubunit binding site corresponding to a low-affinity and a high-affinity binding site of benzodiazepines 35. Thus, distinct modulatory binding sites for benzodiazepines and for VOCs may also exist on the GABA_A_Rs.

Hydrophobicity, however, is a common feature of both substance classes. A hydrophobic nature is a requirement for the VOCs and would enable them to reach their targets in the brain. Only a few reports are available that demonstrate and discuss the route of volatile odorants in vivo besides their binding to the olfactory receptors in the nasal epithelium 36–38. Odorant receptors are G-protein- coupled receptors that lead to an enhancement of cAMP in the cell, which triggers downstream signaling cascades 15,39.

These modulatory sites could have similar physiological consequences. Carvacrol, for example, was identified to harbor anxiolytic properties using an animal model of anxiety, the elevated plus maze test. In contrast, no effect on locomotor activity was observed for carvacrol. Mice pretreated with carvacrol displayed an increase in latency for the development of convulsions, however, only when used at high concentrations and less effective than (-)-borneol or citral 36,37. Nevertheless, the concentrations of volatile odorants that indeed are present at CNS neurons are still unknown and need further studies for clarification of this issue.

Alcohols and anesthetics also modulate GABA_A_Rs and other members of the Cys-loop receptor family 40,41. In vitro mutagenesis as well as X-ray crystallography of a bacterial homolog of the GABA_A_Rs GLIC, isolated from *Gloeobacter violaceus*, implied that the motion of residues in the transmembrane regions 2, 3, and 4 enable a transient communication between the inter- and intrasubunit cavities for propofol and other anesthetics 41,42. The crystallization of GLIC in the absence and presence of ethanol intriguingly illustrated residues localized in the transmembrane cavities involved in ethanol binding. F238, for example, participates in the binding of ethanol and similar modulators, thereby stabilizing the open-state conformation 41. Terpene structures that were able to modulate GABA_A_Rs are characterized by a cyclic structure and the presence of a hydroxyl group, which seem to be required for modulation. Isopulegol, verbenol, and myrtenol were identified to harbor the highest potential of modulation on α1β2 as well as α1β2γ2 GABA_A_Rs. Therefore, we postulate that they may have a similar mechanism to the action of various alcohols or anesthetics via an interaction at the GABA_A_Rs transmembrane domains. The enhancement of GABAergic currents mediated by VOC modulation at GABA_A_Rs might be explained by conformational changes following modulator interaction resulting in stabilization of the open channel.

In summary, we have identified some distinct volatiles, specifically terpenes, as modulators of the GABA_A_Rs providing an explanation of sedative effects. It was demonstrated by Löw *et al*. that in contrast to α1-containing receptors α2- and α3-containing receptors mediate anxiolytic effects 6. If terpenes act in a subunit-specific manner or act nonspecifically, it still needs further investigation. Recordings from GABA_A_Rs expressing neurons or brain slices might also prove useful to investigate the role of these substances in the target neuronal tissue. Thus, terpenes with distinct structural properties may mediate sedative or anxiolytic mechanisms involving GABA_A_Rs.

## Abbreviations

DCM: Dichloromethane
GABA_A_Rs: GABA receptors
HEK: human embryonic kidney
